# Association between Dopamine D4 Receptor Polymorphism and Age Related Changes in Brain Glucose Metabolism

**DOI:** 10.1371/journal.pone.0063492

**Published:** 2013-05-22

**Authors:** Nora D. Volkow, Dardo Tomasi, Gene-Jack Wang, Frank Telang, Joanna S. Fowler, Rita Z. Goldstein, Nelly Klein, Christopher Wong, James M. Swanson, Elena Shumay

**Affiliations:** 1 National Institute on Drug Abuse, National Institutes of Health, Bethesda, Maryland, United States of America; 2 National Institute on Alcohol Abuse and Alcoholism, National Institutes of Health, Bethesda, Maryland, United States of America; 3 Medical Department Brookhaven National Laboratory, Upton, New York, United States of America; 4 Department of Pediatrics University of California Irvine, Irvine, California, United States of America; Sanjay Gandhi Medical Institute, India

## Abstract

Aging is associated with reductions in brain glucose metabolism in some cortical and subcortical regions, but the rate of decrease varies significantly between individuals, likely reflecting genetic and environmental factors and their interactions. Here we test the hypothesis that the variant of the dopamine receptor D4 (*DRD4*) gene (VNTR in exon 3), which has been associated with novelty seeking and sensitivity to environmental stimuli (negative and positive) including the beneficial effects of physical activity on longevity, influence the effects of aging on the human brain. We used positron emission tomography (PET) and [^18^F]fluoro-D-glucose (^18^FDG) to measure brain glucose metabolism (marker of brain function) under baseline conditions (no stimulation) in 82 healthy individuals (age range 22–55 years). We determined their DRD4 genotype and found an interaction with age: individuals who did not carry the 7-repeat allele (**7R−**, n = 53) had a significant (p<0.0001) negative association between age and relative glucose metabolism (normalized to whole brain glucose metabolism) in frontal (r = **−**0.52), temporal (r = **−**0.51) and striatal regions (r = **−**0.47, p<0.001); such that older individuals had lower metabolism than younger ones. In contrast, for carriers of the 7R allele (**7R+** n = 29), these correlations with age were not significant and they only showed a positive association with cerebellar glucose metabolism (r = +0.55; p = 0.002). Regression slopes of regional brain glucose metabolism with age differed significantly between the **7R+** and **7R−** groups in cerebellum, inferior temporal cortex and striatum. These results provide evidence that the DRD4 genotype might modulate the associations between regional brain glucose metabolism and age and that the carriers of the 7R allele appear to be less sensitive to the effects of age on brain glucose metabolism.

## Introduction

Aging of the human brain is associated with decreases in brain glucose metabolism [1]. However, there is significant inter-individual variability in the age associated decreases in regional brain glucose metabolism [2]. This variability is likely to reflect both genetic and environmental factors and their interactions. The effects of aging on brain glucose metabolism have been mostly investigated with respect to the apolipoprotein E gene (*APOE* ε4). Several studies have corroborated that healthy individuals carriers of the ε4 allele, which increases the risk of Alzheimer’s disease (AD), have lower regional cerebral glucose metabolism than non-carriers in brain regions where glucose metabolism is markedly reduced in AD patients (posterior cingulate, precuneus, parietal, temporal, and prefrontal cortex) [3], [4], [5], [6].

Although personality traits [7], [8] have been linked to health and disease (e.g., smoking and pulmonary disease, inactivity and cognitive decline), the effects of variations in the genes influencing personality on aging of the human brain have not been thoroughly investigated. Since heritability studies clearly point to a strong genetic component underlying human personality [9], it is reasonable to expect that genes influencing personality are likely to contribute to the effects of aging in the human brain.

Here we tested the hypothesis that the dopamine D4 receptor (*DRD4*) gene, which is associated with the personality trait of novelty seeking [1], [10], [11], and with higher sensitivity to both positive and negative environmental exposures [12], [13], [14], influences the effects of aging on the human brain. Indeed, we recently showed that the DRD4 modulated the beneficial effects of enriched environments on longevity both in humans and in laboratory animals [15]. Moreover, an allele in the *DRD4 gene* was found to influence activity in the anterior cingulate gyrus [16], which is one of the brain regions most sensitive to the effects of age on brain glucose metabolism [17].

The *DRD4* gene contains a 48-bp variable number tandem repeat (VNTR), located in exon 3, that encodes the third intracellular loop of the receptor [18], [19]. Alleles containing from 2 to 11 repeats (2R to 11R) have been identified, with the three most common variants (2R, 4R and 7R) accounting for over 90% of the population’s allelic diversity [20]. The D4 receptor 7R variant displays decreased sensitivity to DA compared to the D4 receptor 4R variant [21] and differs from the 4R variant in that it does not form heteromers with D2 receptors in the striatum [22], [23]. Based on our previous observations that reductions in striatal D2 receptors with aging were associated with decreases in metabolism in frontal and temporal cortices [24] we hypothesized that individuals carrying the 7R variant, which does not heteromerize with the D2R, will not show the age-related metabolic decreases in frontal and temporal brain regions.

To test this hypothesis we used Positron Emission Tomography (PET) and 2-deoxy-2-[^18^F]fluoro-D-glucose (^18^FDG) to compare the effect of age on brain glucose metabolism between 7R-carriers (individuals with 4/7, 7/7, 2/7 and 5/7 genotypes), referred to as **7R+** and non-carriers of the 7R-allele (individuals with 4/4, 2/4, 4/5 and 4/6 genotypes), herein referred to as **7R−**.

## Results

There were no demographic differences between **7R−** and **7R+** individuals (Table 1). However, the groups differed in the personality measures of Achievement and Self-control. Scores on Achievement were significantly higher in **7R+** (15±3) than **7R−** individuals (12±5) (p<0.05) and those on Self-control were also higher in **7R+** (19±3) than **7R−** individuals (15±5)(p<0.01). There were no differences in the scores of PEM between **7R−** (50±10) and **7R+ (**53±8).

**Table 1:** Demographic characteristics of individuals without the 7R allele (**7R−**) and those with the 7R allele (**7R+**).

	7R− (n = 53)	7R+ (n = 29)
Age	35.4±9	35.8±8
BMI	25±4	25±3
Gender	21 F, 32 M	11 F, 18 M
**Ethnicity**		
AA	17	14
C	23	10
Others	13	5
IQ	100±11	102±9
SES	36±12	38±14
PEM	50±10	53±8
§Achievement	12±5	15±3*
§Control	15±5	19±3**
§Beck	3±5	4±5

* Differences between groups *p<0.05; ** 0.001.

§ Personality measures were obtained in 50/53 **7R−** and 27/29 **7R+**. AA = African American, C = Caucasian, Others = Asian, Native American.

Whole brain glucose metabolism did not differ between **7R−** (39.9±6 µmol/100 g/min) and **7R+** individuals (39.2±6 µmol/100 g/min). The correlation between age and whole brain glucose metabolism was not significant neither for the **7R−** (r = −0.07) or the **7R+** group (r = **−**0.21).

In order to test if there were regions in the brain were glucose metabolism differed between the genotypes we performed an SPM comparison of the normalized glucose metabolic images between the groups (**7R−** vs **7R+**). This analysis revealed no significant differences; indicating no effect of genotype on regional brain glucose metabolism.

**Figure 1:**
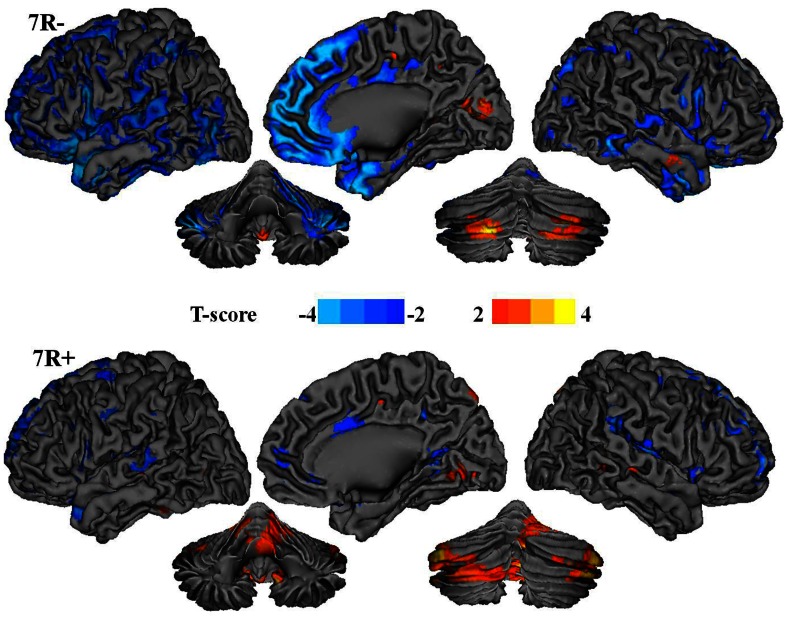
SPM results for the effects of age on brain glucose metabolism (p<0.001, uncorrected) shown on a surface rendering of the brain and of the cerebellum for the individuals without the 7R allele (7R−) and for the individuals with the 7R allele (7R+). Negative correlations are displayed in blue and positive correlation in red.

To assess if there were differences in the effects of age on regional brain glucose metabolism between the individuals with a **7R−** and those with a **7R+** we assessed the interaction effect between Age and Genotype. This analysis revealed that the “interaction” of Age x Genotype was significant, revealing more extensive negative associations between regional brain glucose metabolism and age for **7R−** than for **7R+** individuals (**Figure 1**). Specifically, SPM identified 5 clusters (brain areas) that showed significant negative associations between relative glucose metabolism and age for **7R−** individuals whereas in **7R+** individuals only 1 cluster (brain area) showed a significant positive association (Table 2). The **7R−** individuals showed lower relative glucose metabolic measures with age in frontal regions including medial frontal (BA 10 and BA 11) and superior frontal cortices (BA 8); temporal regions including temporal pole (BA 38), middle and inferior temporal cortices (BA 21 and 20) and insula (BA 13); and also in striatum, fusiform and parahippocampal gyrus (BA 35 and 36) and in cerebellum. In contrast in **7R+** individuals only the cerebellum revealed a positive association with age such that older individuals had greater relative glucose metabolism than younger ones.

**Table 2:** SPM results showing the clusters (brain areas) where brain glucose metabolism was significantly correlated with age for individuals without a 7R allele (**7R−**) (all correlations were negative) and for individuals with a 7R allele (**7R+**) (correlation was positive) along with the regions within the cluster (Gyrus and Brodman Area (BA)), the MNI stereotactic coordinate for center voxel (x,y,z), the T scores and size of the clusters (k voxels).

7R− Individuals Negative Correlations						
Region	BA	K [voxels]	X [mm]	Y [mm]	Z [mm]	T-score
Superior medial frontal	10	2636	4	62	10	7.2
Superior medial frontal	8		**−**2	40	48	5.55
Superior medial frontal	10		**−**2	52	26	5.32
Temporal pole	38	2947	**−**44	14	**−**16	6.3
Insula	13		**−**46	18	**−**4	5.39
Caudate			**−**10	10	8	4.89
Middle temporal	21	565	50	**−**4	**−**20	5.71
Middle temporal	20		48	6	**−**32	5.2
Temporal pole	38		38	14	**−**16	3.97
Parahippocampal	35	402	14	**−**2	**−**32	4.84
Fusiform	36		28	6	**−**48	4.61
Inferior temporal	20	308	**−**56	**−**30	**−**28	4.18
Cerebellum			**−**40	**−**38	**−**32	4.11
Fusiform	20		**−**46	**−**28	**−**28	4.05
**7R+** Individuals Positive Correlations						
Cerebellum		1000	14	**−**62	**−**50	3.67
Cerebellum			16	**−**70	**−**24	3.29
Cerebellum			12	**−**50	**−**18	3.27

All values were significant (p<0.05) after FDR correction (PcCluster) and when applying the more conservative family-wise error FWE correction (PcFWE). Empty cells in the **BA** column indicate that there are no Brodman Areas in these regions. Empty cells in the **k** column indicate that those regions belong to the same cluster as the one indicated by the voxel numbers above them.

**Figure 2:**
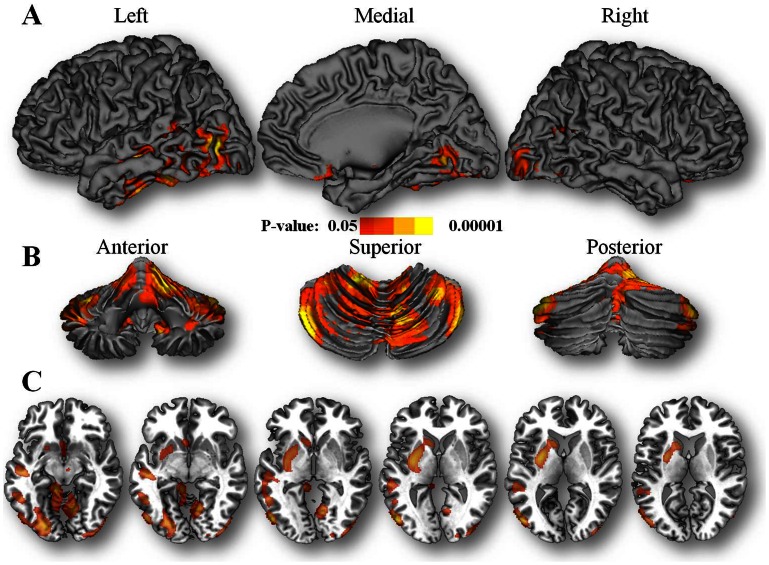
SPM results for the comparisons of the slopes (relative glucose metabolism and age) between the individuals without the 7R allele (7R−) and for the individuals with the 7R allele (7R+). **A.** Shows the results in a surface rendering of the brain; **B.** Shows the results in a surface rendering of the cerebellum; and **C.** Shows the results in axial planes at the level of basal ganglia.

**Figure 3:**
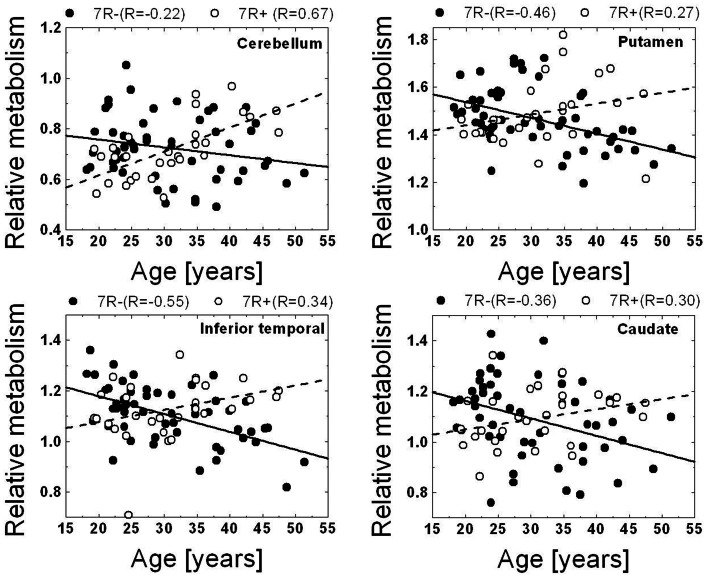
Regression slopes (relative glucose metabolism and age) in cerebellum, putamen, caudate and in inferior temporal cortex (obtained using volumes centered in coordinates from the SPM results) for individuals without the 7R allele (7R−) and for individuals with the 7R allele (7R+).

To determine if the regression slopes between regional brain glucose metabolism and age differed significantly between the groups (**7R−** vs **7R+**) we performed a voxel-wise SPM comparison of age-related changes in relative brain glucose metabolism between **7R−** and **7R+** individuals. This analysis, which compared the slopes between the two groups identified significant differences between them (Figure 2, Table 3). Specifically the slopes showed negative correlations for **7R−**individuals but not for **7R+** individuals in cerebellum, inferior temporal cortex, putamen and caudate (Figure 3).

**Table 3:** SPM results showing the areas were the regression slopes between metabolism and aging differed between the groups (age related decrements **7R−** >**7R+**) along with the regions within the cluster (Gyrus and Brodman Area (BA)), the MNI stereotactic coordinate for center voxel (x,y,z), the T scores and the size of the clusters (k voxels).

Region	BA	K [voxels]	X[mm]	Y [mm]	Z [mm]	T-score
Cerebellum		4333	**−**50	**−**70	**−**32	4.05
Inferior Temporal	20		**−**48	**−**30	**−**28	4.04
Inferior Temporal	20		**−**40	**−**8	**−**46	3.99
Cerebellum		1453	52	**−**70	**−**34	3.72
Cerebellum			36	**−**48	**−**30	3.44
Cerebellum			14	**−**62	**−**50	3.34
Cerebellum		2990	**−**16	**−**40	**−**20	3.64
Cerebellum			**−**8	**−**40	**−**10	3.45
Cerebellum			14	**−**64	-12	3.18
Putamen			**−**28	**−**2	6	3.5
Caudate			**−**14	16	8	2.8
Midbrain			0	**−**16	**−**16	2.5

All values were significant (p<0.05) after FDR correction. Empty cells in the **BA** column indicate that there are no Brodman Areas in these regions. Empty cells in the **k** column indicate that those regions belong to the same cluster as the one indicated by the voxel numbers above them.

To corroborate the findings from SPM we also did an independent analysis by extracting predefined regions of interest (ROI), which were based on the Talairach brain atlas [25]. These independently extracted ROI analysis corroborated the findings from SPM. Specifically it showed significant relative decreases with age throughout the frontal cortex and most of the temporal cortical regions and in striatum (caudate and putamen) for **7R−** individuals whereas none of the correlations with age in these brain regions were significant for **7R+** individuals (Table S1). It also showed that in **7R+** individuals cerebellar glucose metabolism was positively correlated with age.

Since the **7R−** group had a larger sample than the **7R+** group (52 vs 29 individuals) we performed a separate analysis that included a subgroup of 29 individuals from the **7R−** that were age and sex matched to the individuals from the **7R+** group. The results obtained for the group comparison with the same sample sizes (n = 29), yielded the same results as for the complete sample or **7R−**. Specifically, in the **7R−** subgroup, age was negatively associated with relative glucose metabolism in superior medial frontal cortex (BA 8 and 10), in temporal pole (BA 38), insula (BA 13), parahippocampal gyrus (BA 35) and in caudate (Figure S1A, Table S2). SPM comparisons of age-related changes in glucose metabolism between **7R−** and **7R+** individuals (comparison of the slopes) showed that the slopes differed in cerebellum, inferior temporal cortex, putamen and caudate (Figure S1B).

The groups (**7R−** vs **7R+**) consisted of African Americans and Caucasians, so to determine if there were differences on the effects of age on brain glucose metabolism as a function of ethnicity we performed SPM comparisons for the regression slopes between age and brain glucose metabolism between African Americans and Caucasians. This analysis showed that the only significant difference was greater age effects in right cerebellar glucose metabolism in African Americans than in Caucasians (x,y,z = 34, **−**48, **−**34, T = 3.5, p = 0.02, cluster corrected). However, the independent ROI analysis did not corroborate this (African American r = 0.14; Caucasians r = 0.03).

## Discussion

These findings corroborate our hypothesis that the DRD4 might modulate the effects of aging on regional brain glucose metabolism and identifies reduced effects of age in individuals with the 7R allele (**7R+**) compared to individuals without it (**7R−**). The effects of age on whole brain glucose metabolism did not differ between **7R+** and **7R−** individuals, which indicate that the genotype by age interaction was regionally specific. In **7R−** individuals relative glucose metabolism was negatively correlated with age in frontal and temporal brain regions, such that older subjects had lower glucose metabolic values than the younger ones. These regional brain glucose metabolic effects of age are consistent with our prior findings in the general population [26]. These results are also consistent with our recent findings showing that in humans the 7R+ is associated with longevity and with increased physical activity and that DRD4 knockout mice do not show the increases in longevity observed in wild type mice when they are exposed to enriched environments (social and physical) [15].

Using dual PET tracers ([^11^C]raclopride and ^18^FDG) we showed that glucose metabolism in frontal and temporal cortices and in caudate was associated with striatal D2 receptor availability [27]. Moreover, in separate studies of aging we showed that the age-associated reductions in frontal and temporal glucose metabolism were associated with reduced availability of dopamine D2 receptors in striatum [24]. This suggests an involvement of striatal D2 receptors in the changes in frontal and temporal (also striatal) glucose metabolism that occur with aging [28]. Noteworthy, D4 receptors translated from the common 4R variant but not the 7R variant, form heteromers with D2 receptors [22], [23]. In the striatum D4 receptors co-localize with D2 receptors in corticostriatal glutamatergic terminals and activation of the D2–D4 receptor heteromer potentiates D4 receptor mediated inhibition of glutamate release, which does not occur in the D4 receptor with the 7R variant [23]. Since neuronal and glial glucose metabolism is activated by glutamate [29], [30] it is plausible that the enhanced corticostriatal glutamatergic transmission in D4 receptors with the 7R variant could help counteract age related decreases in regional brain glucose metabolism [31]. Indeed, decreases in glutamate levels in the striatum of elderly individuals have been associated with age-induced cognitive decline [32].

We had previously reported an association between the personality measure of PEM and glucose metabolism in medial and lateral prefrontal and medial and inferior temporal regions [33]. PEM includes measures of Achievement (persistence), which is a trait that favors healthy behaviors that have been associated with healthier aging [34]. Though PEM did not differ between **7R+** and **7R−** individuals the scores on the Achievement scale were significantly higher for **7R+** than **7R−** individuals and so was the score on the Self-control scale. Thus we speculate that a greater sustainability of effort in **7R+** individuals along with their greater self-control could increase their engagement in everyday activities (social, intellectual, physical) that may protect them against the age-associated decline in brain function. Indeed engagement in both physical and social activities has been shown to decrease the rate of cognitive decline with aging [35], [36], [37]. Moreover, we recently showed that elderly individuals (>90 years of age) with the 7R+ allele were more physically active than elderly 7R− individuals [15]. The prefrontal cortices and the striatum, which were regions where the decreases in glucose metabolism with age were reduced in **7R+** individuals, are part of “control” networks in the brain [38], and thus their resilience to aging could contribute to their higher scores in self-control.

In our sample the individuals with 7R+ had higher scores on the measures of Self-control than 7R-individuals. This was surprising since the 7R variant of the *DRD4* gene has been associated with a greater risk of ADHD [39], which is a disorder characterized by impulsivity [40]. However D4 receptor knockout mice do not differ in measures of impulsivity when compared with wild type mice [41], which indicates that reduced D4 receptor signaling (as may occur with the 7R variant) by itself does not mediate the impulsive phenotype. Indeed consistent with our findings a study in college students reported that those with the 7R variant showed greater response inhibition than those without the 7R variant [42]. Moreover in children with ADHD those with the 7R variant do not perform worse in measures of inhibitory control than children without the 7R variant [43].

In **7R+** individuals the effects of age on regional brain glucose metabolism were only significant in cerebellum where glucose metabolism showed a positive correlation with age, whereas the association with age was non-significant in **7R−** individuals. This is consistent with preclinical studies reporting differences in baseline cerebellar glucose metabolism (as well as prefrontal glucose metabolism) between D4 receptor knockout and wild-type mice [44], which would suggest that D4 receptors modulate cerebellar activity. Interestingly, a study in adult ADHD individuals reported that those who carry the 7R allele had smaller volumes in cerebellum and in superior frontal cortex than non-carriers, which correspond to regions where **7R+** individuals did not show the negative association with aging observed in **7R−** individuals in our study of healthy controls. This seemingly paradoxical finding could reflect the overall greater sensitivity of carriers of a 7R allele to both negative consequences of adverse stimuli as well as positive consequences of beneficial stimuli [45], [46], [47], therefore the same genotype may be differentially manifested in ADHD affected children (as a result of adverse environmental effects) than in healthy adults (as a result of favorable environmental effects).

Prior brain imaging studies that evaluated the influence of genes on the effects of age on brain glucose metabolism focused on the apolipoprotein E (*APOE-ε4*) allele, which is a major susceptibility gene for late onset Alzheimer’s disease [48]. Those studies reported significantly lower metabolism in older individuals carriers of the *APOE-ε4* variant that in non-carriers in posterior cingulate cortex, precuneus and parieto-temporal cortices [2], [5]. The brain regions that differed as a function of the *APOE-ε4* have little overlap with the brain regions where we show differences on the effects of age on the basis of *DRD4* genotype. This suggests that these genotypes have distinct effects on the effects of age on regional brain glucose metabolism. However, future studies with larger samples should assess potential interactions between the DRD4 and the APOE genotype.

Limitations for this study include the following: It is a cross sectional study whereas a longitudinal design would have allowed us to assess the extent to which *DRD4* variants regulate the rate of brain metabolic changes with age and to address the limitation from the few data point per age group. It does not relate the changes in regional brain glucose metabolism with measures of cognitive function or personality and thus we cannot determine their functional significance. However, the range of values for the brain regions where the slopes with age differed between groups (differences between low and high 43–50%) is within the range reported for differences in regional brain glucose metabolism in patients with Alzheimer’s disease (25% lower compared to controls) [49], [50] and thus likely to be functionally meaningful. It is possible that the differential effects of the *DRD4* alleles vary by gender or ethnicity but our study sample size does not allow us to assess this. However, the lack of differences on the effects of age on the regional brain glucose metabolic measures between African Americans and Caucasians (only difference in right cerebellum, which was not corroborated by ROI) suggests that it is not influenced by ethnicity. The groups had unequal sample sizes; the **7R−** being larger than **7R+** (where most effects were not significant). However the analysis on a matched subset of 7R**−** individuals with the same sample sizes for both group (n = 29) yielded the same results as obtained with the complete sample. Also we report slope differences for the effects of age on relative glucose metabolism between **7R+** and **7R−** but we cannot determine if this reflects higher glucose metabolism in frontal and temporal cortices in younger **7R−** individuals rather than accelerated aging in older **7R−** individuals. Finally, we show significant differences in the correlation between age and brain glucose metabolism, but correlations do not necessarily imply a causal association thus future studies investigating the factors that underlie these correlations might be more informative *vis á vis* the role of the DRD4 gene in these changes.

## Materials and Methods

### Ethics Statement

The studies were approved by the Committee on Research Involving Human Subjects at Stony Brook University. After explaining the procedure, written informed consent was obtained from each subject.

### Subjects

We studied 82 healthy controls; of which 53 individuals (65%) (35.4±9 years of age; 21 F, 32 M) did not carry a 7 allele (**7R−**) (34 had 4/4, 15 had 2/4, 2 had 4/5 and 2 had 4/6 genotypes) and 29 individuals did (**7R+**) (35%) (35.8±8 years of age; 11 F, 18 M) (23 had 4/7, 3 had 7/7, one had 2/7, one had 5/7 and one had 6/7 genotypes). These individuals had participated as control subjects for PET studies that measured brain glucose metabolism at baseline (no stimulation, eyes open, dimly lit quiet room). They were recruited using public advertisement seeking healthy volunteers, who were initially screened by phone and subsequently evaluated for eligibility by a physician. Subjects were excluded if they had current or past psychiatric disorders (including drug abuse or dependence), neurological diseases, significant medical illnesses, were currently on medication(s) (including over the counter drugs) or were pregnant. As part of the evaluation procedure, subjects had a physical, psychiatric and neurologic examination and completed a neuropsychological battery to ensure that there was no evidence of cognitive impairment. Routine laboratory tests were performed as well as a urine test to rule out the use of psychoactive drugs. Subjects were instructed to discontinue any over-the-counter medications two weeks prior to the PET scan and to refrain from drinking alcohol the week prior to the PET scan. Cigarettes, food, and beverages (except for water) were discontinued at least 4 hours prior to the study.

### Personality Measures

Participants completed the multidimensional personality questionnaire (MPQ) [51]. The personality measures were scored for the personality dimension of Positive Emotionality (PEM: extraversion), and for the measures of Achievement (ambitious, enjoys effort, likes challenging tasks, persistent, works hard) (which constitutes a subscale within the Positive Emotionality Superfactor) and Self-control (careful, plans ahead, reflective, rational, organized, tries to anticipate events). These measures were selected since they have been associated with health outcomes [7], [52].

### DNA Extraction and Genotyping

Genomic DNA was extracted from venous blood cells using PAXgene Blood DNA kit (Qiagen, Germantown, MD, USA) according to the manufacturer’s protocol. Genotyping of the 48 bp VNTR was performed by PCR with the following primers: forward: 5′-AGGACCCTCATGGCCTTG-3′ and reverse: 5′- GCGACTACGTGGTCTACTCG-3′. Amplification was performed using Platinum High Fidelity polymerase system (Invitrogen, Life Technologies, NY, USA). PCR products were resolved using QIAxcel Capillary Electrophoresis (Qiagen, Germantown, MD, USA) and genotypes were assigned according to the amplicons length. To ensure the correctness of the genotyping results, half of the samples were tested in duplicates and triplicates.

### PET Studies

We used an HR+ tomograph (resolution 4.5×4.5×4.5 mm full width half-maximum, 63 slices) in 3D dynamic acquisition mode. Subjects were scanned under baseline conditions with ^18^FDG as described elsewhere [53]. Briefly, a 20 minutes emission scan was started 35 minutes after injection of 4–6 mCi of ^18^FDG, and arterialized blood was used to measure ^18^FDG in plasma. During the uptake period of ^18^FDG subjects were resting (no stimulation) in a quiet dimly lit room (eyes open) with a nurse by their side to ensure that they did not fall asleep. Thirty minutes after ^18^FDG injection subjects were positioned in the PET scanner. Metabolic rates were computed using an extension of Sokoloff’s model [54]. The emission data for all the scans were corrected for attenuation and reconstructed using filtered back projection. The regional measures of glucose metabolism were normalized to the average measure of whole brain glucose metabolism (region/whole brain); this was done to enhance the sensitivity of the brain glucose metabolic measures to regional differences; the rationale being that the intersubject variability in whole brain glucose metabolism is larger than the intrasubject variability between brain regions [55].

### Image Analysis and Statistics

The normalized glucose metabolic images were first analyzed using Statistical Parametric Mapping (SPM) [56] and findings were then corroborated with independently drawn regions of interest (ROI). For SPM analysis, the glucose metabolic images were spatially normalized to the stereotactic space of the Montreal Neurological Institute (MNI), which provides 3D stereotaxic coordinates of the location of brain regions [57], [58] **,** using the template provided in the SPM2 package and subsequently smoothed with an 8 mm isotropic Gaussian kernel. One-way analysis of variance (ANOVA) in SPM2 was used to assess differences in brain glucose metabolism between **7R+** and **7R−** subjects. Correlations between relative glucose metabolism and age were based on voxel-wise multiple regression analyses in SPM2. Specifically, three regressors were used to contrast differences in age effects on brain glucose metabolism: a constant regressor capturing the average brain glucose metabolism across subjects and two zero-mean regressors, one for the age of **7R+** subjects and the other for the **7R−** subjects. The threshold of significance was set to p_corr_ <0.05, corrected for multiple comparisons at the cluster level with the random field theory [59].

Brain areas (defined as “clusters” by SPM) showing group × age interaction effects on glucose metabolism were further evaluated by computing for each individual the metabolic values in the center of the clusters using 10 mm^3^ volumes (125 voxels). The Primer of Biostatistics software package [60] was used to compare the linear regression of glucose metabolism with age between **7R+** and **7R−** subjects.

To corroborate the SPM findings, we conducted an independent ROI-based analysis using ROIs extracted using an automated method based on the standard Talairach atlas, which provides 3D sterotaxic coordinates for the location of brain regions [61]. First, ^18^FDG images were mapped onto the Talairach brain using the SPM99 spatial normalization algorithm. The inverse mapping procedure was used to extract the Talairach coordinates of all voxels for a given anatomical region using the coordinates in the Talairach Daemon database [25]. These anatomically defined ROIs were overlapped voxel-by-voxel onto the SPM normalized PET image. These ROIs were used to corroborate the significance (p<0.05) of the correlations between age and metabolism in the areas identified by the whole brain SPM both for the **7R+** subjects and for the **7R−** subjects. Only findings that were significant both by SPM and by the independently extracted ROIs were considered significant.

One-way analysis of variance (ANOVA) was used to assess differences in the scores for PEM, Achievement and Self-control between **7R+** and **7R−** subjects.

To address potential confounds from the difference in the sample sizes between the groups (**7R+** n = 29 vs **7R−** n = 53) we conducted a separate analysis to do the comparisons in a subsample of the **7R−** subjects (n = 29) matched for age and gender (35.6±9 years of age; 11 F, 18 M) to the **7R+** individuals (35±8 years of age; 11 F, 18 M).

Our samples were not large enough to assess ethnicity by genotype interaction but in order to assess the contribution of ethnicity to the effects of aging on brain glucose metabolism we also compared the regression slopes for aging and brain glucose metabolism between African American (n = 31; **7R+**14, **7R−** 17) and Caucasians (n = 33; 7R+10, 7R**−** 23).

### Conclusion

We report significant differences on the effects of age on regional brain glucose metabolism between **7R+** and **7R−** individuals. This corroborates our working hypothesis that the 7R of the *DRD4* gene is associated with resilience to the regional decreases in brain glucose metabolism that occur with aging. Further work is necessary to determine if the higher scores in personality measures of Achievement and Self-Control, which are traits that promote sustainability of effort and greater engagement in everyday activities, could be one of mechanism that protects **7R+** individuals against the effects of brain aging.

## Supporting Information

Figure S1 **A**. SPM results for the effects of age on brain glucose metabolism (p<0.001) for a subset of **7R−** individuals (n = 29) that were matched for age and gender to **7R+** individuals (p<0.001, uncorrected). Negative correlations are displayed in blue and positive correlation in red. **B**. SPM results shown in axial planes for the comparisons between the comparisons of the slopes (relative glucose metabolism and age) between the subset of **7R−** (n = 29) individuals and the **7R+** (n = 29); contrast shows significantly greater age effects for **7R−** than **7R+**. Results were similar to those obtained with the complete set of **7R−** subjects.
(TIF)

Table S1 **Correlations between regional brain glucose metabolism and aging for 7R− and for 7R+ individuals on the independently extracted ROI.** Data in the cells correspond to “r” and “p” values. All correlation with age were negative except for those identified as positive (+). NS = not significant.
(DOC)

Table S2 **SPM results showing the clusters where brain metabolism was negatively correlated with age for a subgroup of 7R− individuals ( = 29) that were age and gender matched to 7R+ individuals (n = 29).** Table identifies regions within the cluster (Gyrus and Brodman Area (BA)), the MNI stereotactic coordinate for center voxel (x,y,z), the T scores and size of the clusters (k voxels). All values were significant (p<0.05) after FDR correction.
(DOC)
