# Supplementary figures and images for: Association between Dopamine D4 Receptor Polymorphism and Age Related Changes in Brain Glucose Metabolism
Source: PLoS One. 2013 May 22;8(5):e63492. doi: 10.1371/journal.pone.0063492 (PMC3661541; doi:10.1371/journal.pone.0063492)

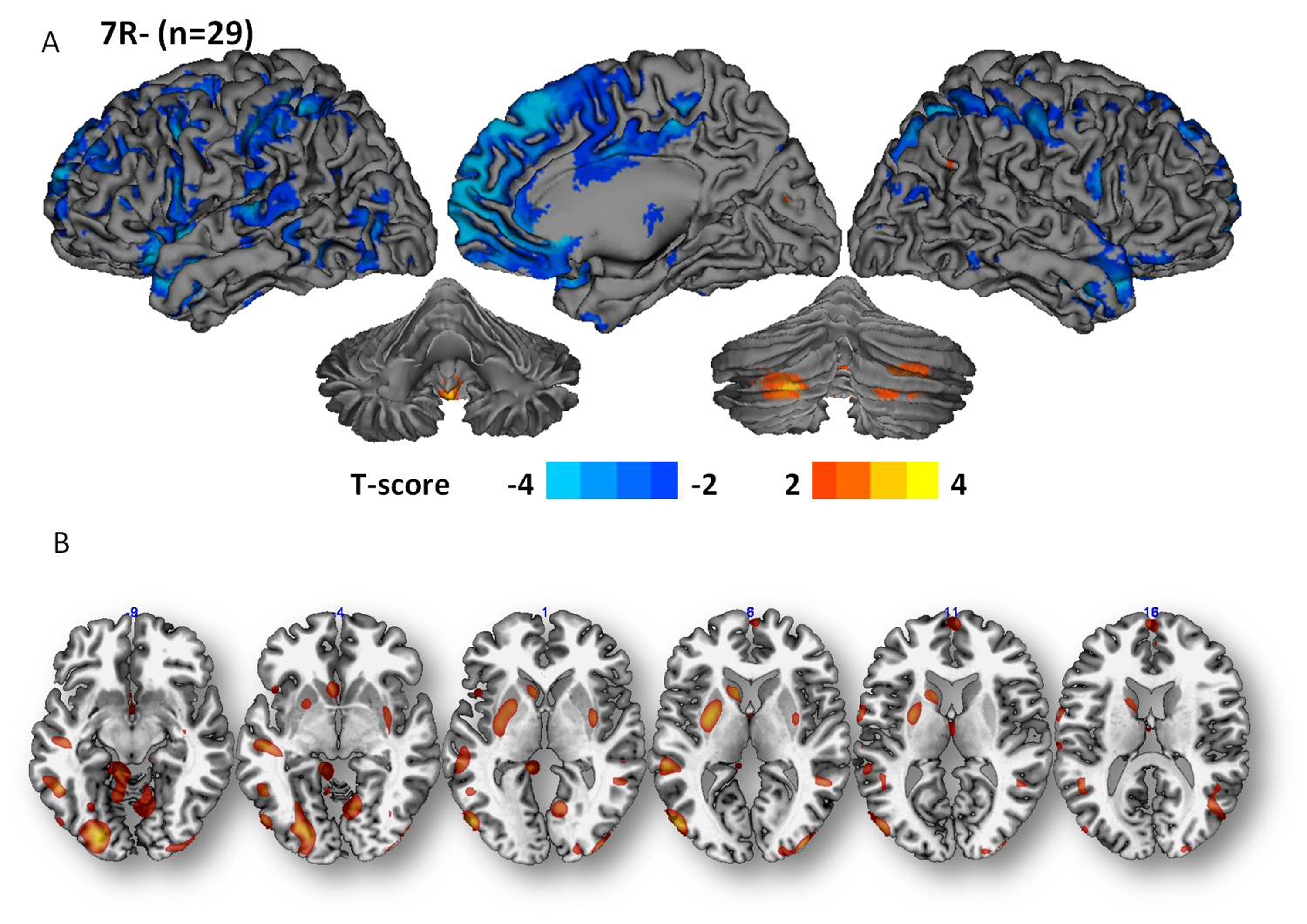

Supplement: Figure S1 — A. SPM results for the effects of age on brain glucose metabolism (p<0.001) for a subset of 7R− individuals (n = 29) that were matched for age and gender to 7R+ individuals (p<0.001, uncorrected). Negative correlations are displayed in blue and positive correlation in red. B. SPM results shown in axial planes for the comparisons between the comparisons of the slopes (relative glucose metabolism and age) between the subset of 7R− (n = 29) individuals and the 7R+ (n = 29); contrast shows significantly greater age effects for 7R− than 7R+. Results were similar to those obtained with the complete set of 7R− subjects. (TIF) [file pone.0063492.s001.tif]
