# Supplementary material for: Association between Dopamine D4 Receptor Polymorphism and Age Related Changes in Brain Glucose Metabolism
Source: PLoS One. 2013 May 22;8(5):e63492. doi: 10.1371/journal.pone.0063492 (PMC3661541; doi:10.1371/journal.pone.0063492)
Supplement: Table S1 — Correlations between regional brain glucose metabolism and aging for 7R− and for 7R+ individuals on the independently extracted ROI. Data in the cells correspond to “r” and “p” values. All correlation with age were negative except for those identified as positive (+). NS = not significant. (DOC) [file pone.0063492.s002.doc]

|  | **7R-** Individuals (n=53) | | **7R+** Individuals (n=29) | |
| --- | --- | --- | --- | --- |
|  | **Left** | **Right** | **Left** | **Right** |
| Sup Frontal | 0.51; 0.0001 | 0.41; 0.003 | 0.27; NS | 0.33; NS |
| Mid Frontal | 0.45; 0.0007 | 0.23; NS | 0.21; NS | 0.35; NS |
| Inf Frontal | 0.52; 0.0001 | 0.31; 0.03 | 0.21; NS | 0.26; NS |
| Med Frontal | 0.46; 0.0005 | 0.33; 0.02 | 0.18; NS | 0.16; NS |
| Rectal BA 11 | 0.51; 0.0001 | 0.48; 0.0002 | 0.19; NS | 0.18; NS |
| ACC BA 32 | 0.33; 0.02 | 0.39; 0.004 | 0.23; NS | 0.31; NS |
| Sup Temp | 0.58; 0.0001 | 0.32; 0.02 | 0.28; NS | 0.21; NS |
| Mid temp | 0.44; 0.001 | 0.31; 0.03 | 0.08; NS | 0.03; NS |
| Inf temp | 0.41; 0.003 | 0.38; 0.006 | 0.12; NS | 0.07; NS |
| Caudate | 0.43; 0.002 | 0.33; 0.02 | 0.05; NS | 0.27; NS |
| Putamen | 0.47; 0.0004 | 0.34; 0.02 | 0.17; NS | 0.11; NS |
| Cerebellum | 0.25; NS | 0.22; NS | +0.55; 0.002 | +0.51; 0.005 |

Supplemental Table 1. Correlations between regional brain glucose metabolism and aging for **7R-** and for **7R+** individuals on the independently extracted ROI. Data in the cells correspond to “r” and “p” values. All correlation with age were negative except for those identified as positive (+). NS = not significant.
