# Supplementary material for: Association between Dopamine D4 Receptor Polymorphism and Age Related Changes in Brain Glucose Metabolism
Source: PLoS One. 2013 May 22;8(5):e63492. doi: 10.1371/journal.pone.0063492 (PMC3661541; doi:10.1371/journal.pone.0063492)
Supplement: Table S2 — SPM results showing the clusters where brain metabolism was negatively correlated with age for a subgroup of 7R− individuals ( = 29) that were age and gender matched to 7R+ individuals (n = 29). Table identifies regions within the cluster (Gyrus and Brodman Area (BA)), the MNI stereotactic coordinate for center voxel (x,y,z), the T scores and size of the clusters (k voxels). All values were significant (p<0.05) after FDR correction. (DOC) [file pone.0063492.s003.doc]

| **Region** | **BA** | **k [voxels]** | **X[mm]** | **Y[mm]** | **Z[mm]** | **T-score** |
| --- | --- | --- | --- | --- | --- | --- |
| Superior medial frontal | 10 | 2636 | 4 | 62 | 10 | 6.0 |
| Superior medial frontal | 8 |  | -2 | 40 | 48 | 4.2 |
| Superior medial frontal | 10 |  | -2 | 52 | 26 | 4.9 |
| Temporal pole | 38 | 2947 | -44 | 14 | -16 | 5.0 |
| Insula | 13 |  | -46 | 18 | -4 | 4.6 |
| Caudate |  |  | -10 | 10 | 8 | 4.4 |
| Middle temporal | 21 | 565 | 50 | -4 | -20 | 3.7 |
| Temporal pole | 38 |  | 38 | 14 | -16 | 3.8 |
| Parahippocampal | 35 | 402 | 14 | -2 | -32 | 3.5 |

Supplemental Table 2. SPM results showing the clusters where brain metabolism was negatively correlated with age for a subgroup of **7R-** individuals (=29) that were age and gender matched to **7R+** individuals (n=29). Table identifies regions within the cluster (Gyrus and Brodman Area (BA)), the MNI stereotactic coordinate for center voxel (x,y,z), the T scores and size of the clusters (k voxels). All values were significant (p <0.05) after FDR correction.
